# New method for visualization of C-heterochromatin in synaptonemal complex spreads

**DOI:** 10.3897/CompCytogen.v7i2.5187

**Published:** 2013-05-22

**Authors:** Artem P. Lisachov

**Affiliations:** 1 Institute of Cytology and Genetics,Russian Academy of Sciences, Siberian Department & Novosibirsk State University, Novosibirsk 630090, Russia

**Keywords:** Synaptonemal complexes, pachytene, DAPI, C-banding

## Abstract

DAPI staining of the metaphase chromosomes pretreated with barium hydroxide generates a C-like banding pattern. In this work a protocol for visualizing similar pattern at the synaptonemal complex (SC) spreads after immunostaining is suggested. This method was used to visualize centromeric and sex heterochromatin at the SC spreads of guppy fish (*Poecilia reticulata* Peters, 1859). The efficiency of this method was further confirmed at SC spreads of the northern red-backed vole (*Myodes rutilus* (Pallas, 1779)), the guinea pig (*Cavia porcellus* (Linnaeus, 1758)), and the pigmy shrew (*Sorex minutus* Linnaeus, 1766).

## Introduction

Immunofluorescent analysis of synaptonemal complexes (SC) is widely performed in medical and comparative cytogenetics to study chromosome synapsis and recombination in humans with chromosomal abnormalities (Solari 1999) as well as in various plant and animal species (Anderson et al. 1999; Basheva et al. 2010; Borodin et al. 2008). Specific antibodies are used to visualize lateral and central elements of SC, double strand breaks and crossovers. These antibodies are also used to reveal specific meiotic histone modifications and other important characteristics of meiotic cells.

Centromeres are used as an important marker in SC karyotyping. For instance, centromeres are commonly detected by human autoantibodies to human centromere proteins (CREST serum from human scleroderma patients). These proteins are conservative and human antibodies may be used to study various species (Basheva et al. 2010, Del Priore and Pigozzi 2012, Moens 2006). However, their affinity decreases as phylogenetic distance increases. For example, CREST serum from human scleroderma patient can detect centromeres in zebrafish only at concentrations that are five times higher than the concentrations used in mammals (Moens 2006). Centromeres in guppy have not been detected even at higher concentration of human antibodies to human centromere.

In this study a simple protocol was designed to obtain C-like banding on SC spreads and visualize centromeres without the use of antibodies. This method is cheaper and applicable if antibodies fail to detect centromeres because of a large phylogenetic distance. This method is a modified DAPI-staining technique combined with pretreatment commonly used for C-banding. The proposed method was tested on guppy fish. The efficiency of this method was confirmed in three of five examined mammal species. Previously, the DAPI staining after barium hydroxide treatment was shown to visualize C-like banding on the metaphase chromosomes of fish (Russo, Rocco et al. 1999), plants (Barros e Silva and Guerra 2010), insects (Bella and Gosálvez 1991) and human (Heng and Tsui 1993). However, in this study the proposed technique was used for the first time to analyze SCs.

## Materials and methods

Mammal spermatocytes were prepared according to the prescribed method of (Peters et al. 1997). Testicular fragments were immersed in a hypotonic extraction buffer containing 30 mMTris, 50 mM sucrose, 17 mM trisodium citrate dihydrate, 5 mM EDTA, pH 8.2 for 30 to 60 min. A suspension was made in a 40 µl drop of 100 mM sucrose, pH 8.2 (pH was adjusted using NaOH), on a clean glass slide. First, the tubules, approximately 2 cm in length, were torn to pieces between the tips of two fine watchmaker forceps in 20 µl of sucrose solution. Thereafter, the volume was increased to 40 µl and a slightly cloudy suspension was made. The tubular remnants were removed and the remaining suspension was divided between two new clean glass slides that had been dipped just before in a freshly made and filtered (0.2 µM) 1% paraformaldehyde (PFA), pH 9.2, solution containing 0.15% Triton X-100. Nuclei were dried for at least 2 h in a closed box with high humidity. Finally, the slides were washed twice for 2 min in 0.4% Photo Flo (Kodak) and dried at room temperature.

This method also yielded satisfactory results for guppy spermatocytes. However, more accurate results were obtained using the method of (Moens 2006). In brief, 30 µl drops of 1/3×PBS hypotonic solution were placed on the dry slide. The testes of the guppy males were macerated in 50µl to100 µl of PBS, and 1 µl portions of suspension were injected into the drops. The cells were fixed by 1% paraformaldehyde after 20 min of hypotonic treatment. The slides were washed twice for 2 min in 0.4% Photo Flo (Kodak) and dried at room temperature.

The slides with guppy spermatocytes were permeabilized by the incubating in 10 mM sodium citrate solution with 0.1% Tween-20 at 95° C for 20 min before immunostaining was performed. The slides were then cooled to room temperature for 20 min and rinsed in PBS with 0.1% Tween-20 for 2 min twice. Immunostaining was performed according to the protocol described in (Anderson et al. 1999) with slight modifications. SC was detected by rabbit polyclonal antibodies to SC axial element protein SYCP3 (1:500, Abcam, Cambridge) and goat anti-rabbit Cy3-conjugated secondary antibodies (1:500, Jackson, West Grove). The centromeres were detected by human anti-centromere antibodies (ACA) (1:100 for mammals to 1:20 for guppies, Sigma-Aldrich) and goat anti-human FITC and Cy3 (for the vole) conjugated secondary antibodies (1:100, Vector Laboratories). After washing, the antifade solution with DAPI (Vectashield, Vector Laboratories) was mounted on the slides.

The preparations were photographed using Axioplan 2 Imaging microscope with a CCD camera (CV M300, JAI Corporation, Japan), CHROMA filter sets, and an ISIS4 image processing package (MetaSystems GmbH, Germany). The coverslips were carefully removed after the photographs were taken. The preparations were washed in 2×SSC for 5 min to remove the antifade solution and then dehydrated in ethanol series 70%, 80% and 100% for 3 minutes in each. The preparations were then air-dried and stored in 0,2 N HCl at room temperature for 20 min to 30 min. The slides were transferred to saturated barium hydroxide solution at 55°C for 1 min to 10 min. Afterward, the preparations were incubated in 2×SSC at 55°C to 60°C for 60 min. The preparations were re-mounted in the antifade solution with DAPI and the cells were re-photographed.

## Results and discussion

**Figure 1.**
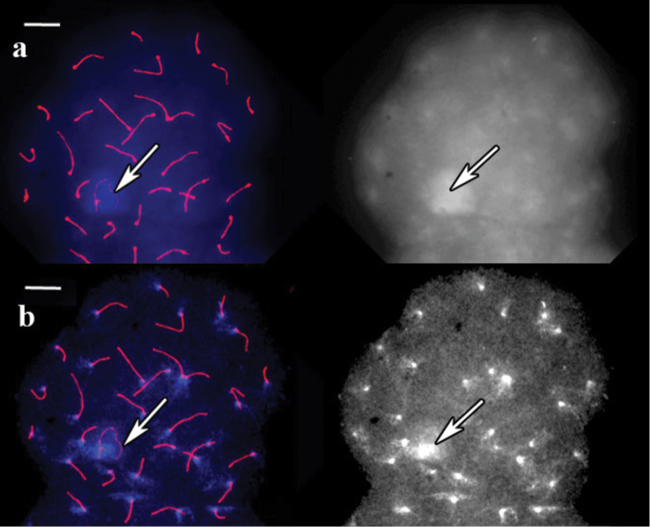
SC spread in red-backed vole. **a** A cell after immunostaining, prior to barium hydroxide treatment. Left: Red shows SCs (long lines) and centromeres (dots), blue represents DAPI. Right: the same image, DAPI channel separately **b** The same cell after 5 min of barium hydroxide treatment. Left: Red shows to SCs (long lines) and centromeres (dots), blue represents DAPI. Right: the same image, DAPI channel separately. Arrows indicate sex bivalent. Scale bars = 20 μm.

In vole, guinea pig and pigmy shrew, the centromeres were marked by antibodies and DAPI signals. In vole, DAPI-stained pachytene cells untreated with barium hydroxide produced a unique banding pattern for each chromosome. This banding pattern is visible even without image contrast adjustment, and bright bands correspond to centromere antibody signals (Fig. 1a). However, the centromeric bands of most chromosomes were indistinguishable by their brightness from the interstitial ones. After the incubation in barium hydroxide for 5 minutes, all the bands except the centromeric bands and the sex heterochromatin faded and disappeared (Fig. 1b).

**Figure 2.**
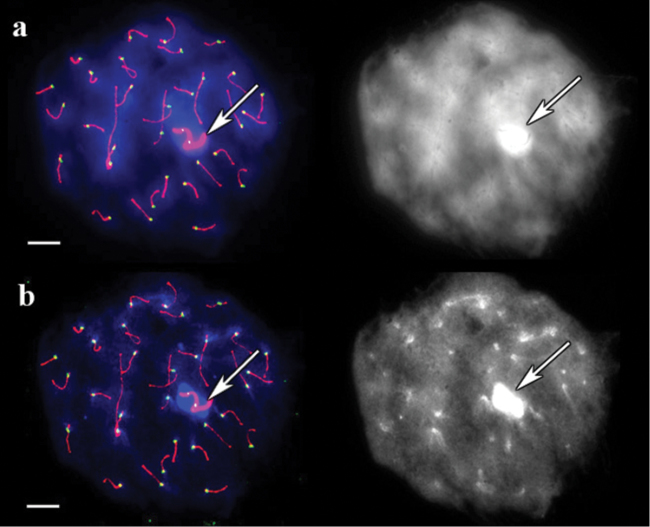
SC spread in guinea pig. **a** A cell after immunostaining, prior to barium hydroxide treatment. Left: Red, green and blue indicate SCs, centromeres, and DAPI, respectively. Right: the same image, DAPI channel separately **b** The same cell after 5 minutes of barium hydroxide treatment. Left: Red, green and blue indicate SCs, centromeres, and DAPI, respectively. Right: the same image, DAPI channel separately. Arrows indicate sex bivalent. Scale bars = 20 μm.

In guinea pig, DAPI-stainied pachytene chromosomes untreated with barium hydroxide produced a weak differential pattern that only became visible after image contrast was adjusted, and the centromeres were indistinguishable (Fig. 2a). After barium hydroxide treatment for 5 minutes, a distinct DAPI signal at the centromeres and over the sex bivalent was observed (Fig. 2b).

**Figure 3.**
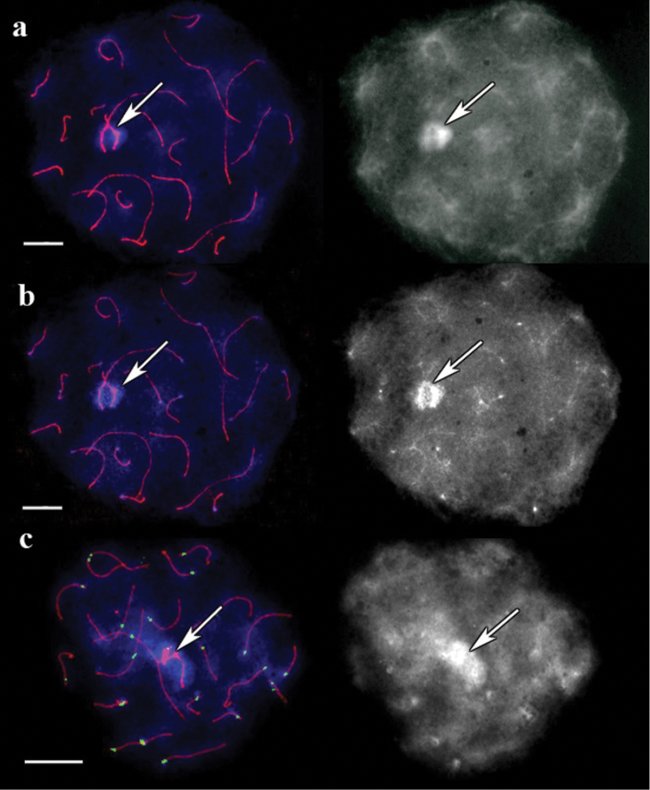
SC spread in pigmy shrew. **a** A cell after immunostaining, prior to barium hydroxide treatment. Left: Red shows SCs, and blue represents DAPI. Right: the same image, DAPI channel separately **b** The same cell after 5 minutes of barium hydroxide treatment. Left: Red shows SCs, and blue represents DAPI. Right: the same image, DAPI channel separately **c** Bright DAPI spots with their corresponding centromere signals. The cell after 5 min of barium hydroxide treatment. Left: Red, green and blue indicate SCs, centromeres, and DAPI, respectively. Right: the same image, DAPI channel separately. Arrows indicate sex bivalent. Scale bars = 20 µm.

In pigmy shrew the DAPI-stained pachytene chromosomes untreated with barium hydroxide produced a unique pattern for each bivalent. This result agrees with that of  (Belonogova et al. 2006). However, the pattern did not exhibit any specific signal at the centromeres (Fig. 3a). Bright DAPI spots were observed after the specimens were treated with barium hydroxide for 5 min (Fig. 3b). Such bright spots corresponded to ACA signals (Fig. 3c). The chromosome-specific DAPI pattern faded but remained recognizable. However, these spots were absent on some bivalents.

In domestic cat (*Felis silvestris catus* (Linnaeus, 1758)) and red fox (*Vulpes vulpes* (Linnaeus, 1758)), the centromeres were successfully detected by ACA, but any centromere-specific DAPI staining was not observed on either untreated pachytene chromosomes, or barium hydroxide-treated chromosomes. The results were not improved although the treatments were modified by increasing HCl pre-incubation time to 30 min, extending barium hydroxide incubation time to 10 min, or decreasing barium hydroxide incubation time to 1 min. The failure to obtain centromeric DAPI signal on the pachytene chromosomes of domestic cat and red fox corresponds to previously published data about the absence of centromeric C-bands and centromeric satellite DNA in cat chromosomes (Matthews et al. 1980; Santos et al. 2004) and absence of centromeric C-bands on most fox chromosomes (Mäkinen 1985).

**Figure 4.**
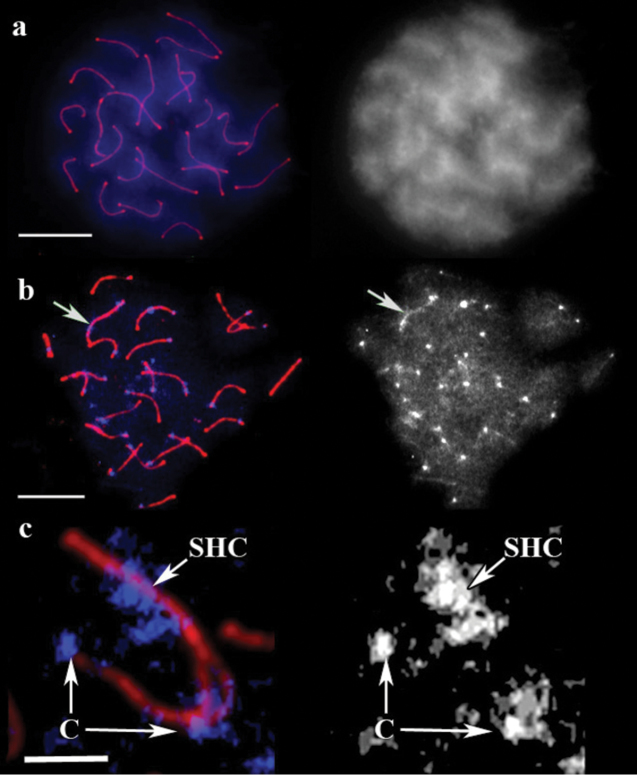
SC spread in guppy. **a** A cell after immunostaining, prior to barium hydroxide treatment. Left: Red shows SCs, and blue represents DAPI. Right: the same image, DAPI channel separately. Scale bar = 20 μm. **b** The cell after 5 min of barium hydroxide treatment. Left: Red shows SCs, and blue represents DAPI. Right: the same image, DAPI channel separately. Arrow indicates sex bivalent. Scale bar = 20 μm. **c** Sex bivalent during pairing. Red shows SC, blue represents DAPI. C: centromere, SHC: sex heterochromatin. Scale bar = 5 μm.

DAPI staining of untreated guppy male pachytene chromosomes produced completely uniform fluorescence without any specific signals (Fig. 4a). ACA did not also show any specific signal in the cell, although the concentration was five times higher than that recommended by the manufacturer. After 5 min of barium hydroxide treatment a bright spot appeared at the end of each bivalent, and the residual fluorescence faded (Fig. 4b). DAPI spots were mostly located at SC ends opposite to the recombination nodules. This finding was attributed to suppressed recombination near the centromeres. This effect has also been described in various vertebrate species including mammals (Basheva et al. 2008) and zebrafish (*Danio rerio* (Hamilton, 1822)) (Moens 2006).

The XY bivalent in guppy was easily distinguishable from a delayed synapsis at early and mid pachytene stages and from an excessive thickening of the axial elements at late pachytene stage. This bivalent showed one small DAPI signal at one end and a large DAPI-positive block close to its opposite end (Fig. 4c). This result is consistent with the pattern obtained by (Nanda et al. 1993) after conventional Giemsa C-banding on guppy metaphase chromosomes. The C-positive bands were detected at the centromeres and at the distal parts of both X and Y chromosomes.

In guppy, staining quality depend on the age of preparations. In this study, optimal results were obtained from two to six month old preparations. Centromeric and sex fluorescent blocks were also visible on fresh slides, but only after image brightness and contrast were adjusted. Although the time allotted for barium hydroxide treatment was extended to 10 min, optimal results from the fresh preparations were not obtained. This may occur possibly because higher level of DNA degradation in the old preparations facilitated denaturation by barium hydroxide.
